# Author Correction: Self-healable polymer complex with a giant ionic thermoelectric effect

**DOI:** 10.1038/s41467-024-44944-6

**Published:** 2024-01-17

**Authors:** Dong-Hu Kim, Zico Alaia Akbar, Yoga Trianzar Malik, Ju-Won Jeon, Sung-Yeon Jang

**Affiliations:** 1 School of Energy and Chemical Engineering, Ulsan National Institute of Science and Technology (UNIST), 50 UNIST-gil, Ulsan, 44919 Republic of Korea; 2 Department of Chemistry, Kookmin University, 77 Jeongneung-ro, Seongbuk-gu, Seoul, 136-702 Republic of Korea; 3 Graduate School of Carbon Neutrality, Ulsan National Institute of Science and Technology (UNIST), 50 UNIST-gil, Ulsan, 44919 Republic of Korea

**Keywords:** Thermoelectrics, Polymers, Conjugated polymers

Correction to: *Nature Communications* 10.1038/s41467-023-38830-w, published online 05 June 2023

In this article the grant number 2022R1A2C1092273 relating to the National Research Foundation of Korea was omitted. The original article has been corrected.

