# Supplementary material for: Self-healable polymer complex with a giant ionic thermoelectric effect
Source: Nat Commun. 2023 Jun 5;14:3246. doi: 10.1038/s41467-023-38830-w (PMC10241813; doi:10.1038/s41467-023-38830-w)
Supplement: Supplementary file 1 — Supplementary Information [file 41467_2023_38830_MOESM1_ESM.pdf]

Supplementary Information

Self-healable Polymer Complex with a Giant Ionic Thermoelectric Effect

Dong-Hu Kim,[1] Zico Alaia Akbar,[1] Yoga Trianzar Malik,[2] Ju-Won Jeon,[2] Sung-Yeon Jang[1,3*]*

[1]School of Energy and Chemical Engineering, Ulsan National Institute of Science and Technology (UNIST), 50 UNIST-gil, Ulsan 44919, Republic of Korea

[2]Department of Chemistry, Kookmin University, 77 Jeongneung-ro, Seongbuk-gu, Seoul 136-702, Republic of Korea

[3]Graduate School of Carbon Neutrality, Ulsan National Institute of Science and Technology (UNIST), 50 UNIST-gil, Ulsan 44919, Republic of Korea

*E-mail: syjang@unist.ac.kr; jwjeon@kookmin.ac.kr

Keywords: ionic thermoelectrics, self-healing, stretchable, ionic thermoelectric capacitor, thermopower

Methods

Materials: 3,4-ethylenedioxythiophene (EDOT), poly(2-acrylamido-2-methyl-1-propanesulfonic acid) (PAAMPSA), phytic acid (PA), poly(ethylene oxide), copper chloride (II) ($CuCl_2$), and ammonium persulfate (APS) were purchased from Sigma-Aldrich and used without further purification. PEDOT:PSS (Clevios P VP AI4083) was purchased from Heraeus.

Measurement of pH: A digital pH meter (Senz pH Pro, Trans Instruments) was used to evaluate the pH values of the PAAMPSA and PEDOT:PAAMPSA solutions (**Supplementary Table 1**). In method 1, equal weights of the PAAMPSA or PEDOT:PAAMPSA solutions (666 mg) were diluted with 40 mL of deionized water to produce equivalent wt. % contents of the PEDOT:PAAMPSA complex in the solutions. In method 2, different weights of the PAAMPSA or PEDOT:PAAMPSA solution were diluted with 40 mL of deionized water to produce equivalent molar concentrations of AAMPSA (2-acrylamido-2-methyl-1-propanesulfonic acid) units (0.012 M) in the solutions.

Characterization of XPS spectra: X-ray photoelectron spectroscopy (XPS) spectra were measured using K-alpha radiation (Thermo Fisher). A PEDOT:PAAMPSA thin film was prepared by spin-coating a PEDOT:PAAMPSA solution onto a Si wafer substrates. The thin film was then heated at 50 °C for 10 min.

Measurement of the ionic Seebeck coefficient: Our home-built system, which was managed using Labview, was used to test the ionic Seebeck coefficient. Peltier devices (40 × 40 × 3.7 mm, TEC1-12706), connected in series with Keithley 2400 (source meter unit), were used to induce a temperature gradient in the sample. A pair of T-type thermocouples (5TC-TT-T-36-72, Omega), connected to a data-acquisition unit (Keysight 34970A), was used to assess the temperature difference between the two electrodes. The thermovoltage output was recorded using a digital multimeter (Keysight 34465A). The ionic Seebeck coefficient of the samples

was obtained from the linear relationship between the thermovoltage and temperature difference from the four experiments with different temperature gradients (ranging from 0.5 °C to 2 °C). All samples were placed in an isolated chamber and evaluated at each RH level (from 70 to 90% RH). For repeated stretching cycle experiments, sample films were prepared on a 3M-VHB-4910 substrate. Identical instruments were used to measure and record the operation of the ITEC device and module with a 10 kΩ load resistor.

Measurement of the ionic and electronic conductivity: An electrochemical impedance spectrometer (EIS; COMPACTSTAT, IVIUM echnologies) was used to evaluate the ionic and electronic conductivity. The amplitude of the AC voltage was 0.1 V, and the frequency ranged from 1 Hz to 250 kHz. The ionic resistance (R_i) and electronic resistance (R_e) are obtained by fitting the Nyquist plot (**Supplementary Fig. 4**) with the equivalent circuit model (**Supplementary Fig. 6**) using the EIS analyzer software. The electrical conductivity was calculated using the following equation:

$$\sigma = \frac{1}{R}\frac{L}{A} \tag{1}$$

R corresponds to the ionic and electronic resistance, L is the distance between the two gold electrodes, and A is the cross-sectional area of the sample film.[1] The thickness of the samples was determined using a surface profiler (KLA Tencor, P6).

Determination of the dielectric constant and loss: The Nyquist plot of each film was recorded using EIS in the same manner as described above. The dielectric constant (ε') and loss (ε'') were calculated using the following equations:[2]

$$\varepsilon' = \frac{Z''}{\omega C_0({Z'}^2 + Z''^2)} \tag{2}$$

and

$$\varepsilon'' = \frac{Z'}{\omega C_0({Z'}^2 + Z''^2)} \tag{3}$$

where ω is the angular frequency (rad s^{-1}), $C_0 = \varepsilon_0 A/d$, Z' is the real impedance, Z'' is the imaginary impedance, and ε_0 is the free-space permittivity (8.854187×10^{-12} F m^{-1}).

Determination of the net ion carrier concentration and net ion diffusion coefficient: The method for determining the net ion carrier concentration (n_i) and ion diffusion coefficient (D_i) was adopted from Bandara's method,[3] which uses a curve-based fitting of the dielectric constant–frequency curve using the following equation:

$$\varepsilon' = \varepsilon'_\infty \left(1 + \frac{\delta}{1+(\omega\tau_m)^B \delta}\right) \quad (4)$$

where ε'_∞ is the high-frequency permittivity, τ_m is the time constant (s), B is the coefficient determined by α ($B = 2[1-\alpha]$), and δ is a dimensionless unit corresponding to the ratio of half the distance between the two electrodes (d in cm) to the Debye length (λ in nm). The fitting of ε' was performed in the middle-linear regime to exclude the experimental error from the left and right ends.[4] The fitting was conducted by plotting ε' as a function of the frequency (on a logarithmic scale). A nonlinear fitting analysis was conducted using Origin© software. n_i and D_i were calculated using the following equations:

$$n_i = \frac{\sigma_i k T \tau_m \delta^2}{(|Z|e)^2 d^2} \quad (5)$$

$$D_i = \frac{d^2}{\tau_m \delta^2} \quad (6)$$

where σ_i is the ionic conductivity (S m^{-1}), k is the Boltzmann constant ($8.617333262145 \times 10^{-5}$ eV K^{-1}), T is the measurement temperature at RT (298 K), $|Z|e$ is the elementary charge of the particle ($1.60217662 \times 10^{-19}$ C), and d is the half-distance (~ 0.2 cm) between the two electrodes.

Characterization of SEM and EDS mapping: Scanning electron microscopy (SEM) and

energy dispersive spectroscopy (EDS) mapping were performed using JEOL JSM 7600F thermal FE-SEM (for self-healing observations and EDS mapping) and S-3400N Normal SEM (for morphology observation). For self-healing observations and EDS mapping, the sample was prepared by drop-casting the PEDOT:PAAMPSA:PA (6.2wt. % PEDOT content) solution onto a glass substrate. The thin films were then heated at 50 °C for 10 min. The PEDOT:PAAMPSA:PA thin film was then scratched along the glass substrate using a razor blade. The cut film was placed inside a humidity-controlled chamber to initiate self-healing. For morphological observations, the samples were prepared by spin-coating the PEDOT:PAAMPSA solution onto a glass substrate. The thin films were then heated at 50 °C for 10 min.

Determination of the thermal conductivity: The thermal conductivity (κ) of PEDOT:PAAMPSA:PA under ambient conditions (25 ℃, ~30% RH) was calculated using the equation:

$$\kappa = C_p \cdot \rho \cdot \alpha \tag{7}$$

where C_p is the specific heat capacity, ρ is the density of the sample, and α is the thermal diffusivity. The thermal diffusivity was measured by laser flash analysis (LFA, NETZSCH) at 30% RH and 25 °C. The thermal diffusivity is described by $\alpha = 0.1388\ d^2/t_{1/2}$, where d is the thickness of the sample, and $t_{1/2}$ is the time required to reach the half-maximum temperature. The specific heat capacity was measured by differential scanning calorimetry (DSC8000, Perkin Elmer). It was obtained using a comparative method based on the following equation:

$$C_p^{test} = \frac{T_{max}^{ref}}{T_{max}^{test}} \cdot \frac{(\rho \cdot d)^{ref}}{(\rho \cdot d)^{test}} \cdot C_p^{ref} \tag{8}$$

where T_{max} is the maximum temperature, ρ is the density, d is the thickness, and the

superscripts ref and test correspond to the reference and test samples, respectively. The C_p was calculated to be 1.897 J g^{-1} K^{-1}, α was determined to be 0.118 mm^2 s^{-1}, and ρ was determined to be 1.454 g cm^{-1}, which produced κ of 0.325 W m^{-1} K^{-1}. The thermal conductivity of the sample was affected by the RH. The κ values of the samples at various RH values were obtained using the following equation:

$$\kappa_t = \kappa_p \o_p + \kappa_w \o_w \tag{9}$$

where $\o$ is the volume fraction, and the subscripts t, p and w correspond to the hydrated PEDOT:PAAMPSA:PA, PEDOT:PAAMPSA:PA at RH 30%, and water, respectively. The κ value of water was 0.6 W m^{-1} K^{-1}. **Supplementary Fig. 11** shows the thermal conductivity of the PEDOT:PAAMPSA:PA (6.2 wt. % PEDOT content) at different RH values. The thermal conductivity slightly decreased after self-healing.

Measurement of the stress-strain characteristics: The stress-strain characteristics of PAAMPSA:PA and PEDOT:PAAMPSA:PA free-standing films were investigated through tensile stress tests using a universal testing machine (Tinius Olsen Model H5K-T) at RT and RH of 60%. The free-standing films were prepared using the solvent casting method. The polymer solution was poured into a rectangular silicon mold to obtain 35 × 5 mm free-standing film with a thickness of ~ 0.8 mm. The strain rate was set to 5 mm $min^{-1,}$ and stretching was continued until the free-standing films ruptured. The free-standing films were held by a pneumatic grip. The gauge length of the films was set at 10^{-11} mm.

Determination of the power output and energy density: The power output (P) and energy density (E) of the PEDOT:PAAMPSA:PA (6.2wt. % PEDOT) ITEC device and ITEC module on stage II were calculated based on the following equations:

$$E = \frac{1}{S} \int V_{out} I dt \tag{10}$$

$$P = \frac{1}{S}\frac{\int V_{out} I dt}{\Delta t} = \frac{1}{S}\frac{\int \frac{V_{out}^2}{R_{load}} dt}{\Delta t} \tag{11}$$

where S is the area of the electrode, V_{out} is the voltage in stage II, R_{load} is the load resistance, and Δt is the duration of stage II.

Supplementary Note 1. Reformulation of Han's theory

The ionic Seebeck coefficient (S_i) of iTE materials with two types of carriers is determined by the following equation:

$$S_i = \frac{\Sigma q S_i^* n_i D_i}{\Sigma q^2 n_i D_i} \tag{12}$$

where q is the electric charge of the ion, n_i is the bulk ion-carrier concentration, D_i is the diffusion coefficient, and S_i^* is the Eastman entropy of each ion.[5] Note that σ_i of a monovalent ion system is determined by the following equation:

$$\sigma_i = n_i \cdot D_i \cdot \frac{e^2}{kT} \tag{13}$$

where e is elementary charge ($e = 1.602 \times 10^{-19}$ C), and k is the Boltzmann constant. S_i is therefore given by

$$S_i = \frac{\Sigma q S_i^* \sigma_i}{\Sigma q^2 \sigma_i} \tag{14}$$

Because σ_i is reciprocal to the ionic resistance (R_i), we can rearrange the equation as follows:

$$S_i = \frac{\Sigma \frac{q S_i^*}{R_i}}{\Sigma \frac{q^2}{R_i}} \tag{15}$$

Since S_i (note that S_i indicates a thermovoltage at a certain temperature difference) of an iTE material with one type of ion (S_x) can be expressed as $S_x = S_i^* \cdot q^{-1}$, then, using Ohm's law ($\frac{V}{R} = I$),

$$S_i = \frac{\Sigma q I_i}{\Sigma \frac{q}{R_i}} \tag{16}$$

where $I_i = \frac{S_i^*}{q R_i}$ is the ionic thermocurrent per unit temperature difference for each ion. For a monovalent system, $q_+ = -q_-$. Thus, S_i can be abbreviated as

$$S_i = \frac{\Sigma I_i}{\Sigma \frac{1}{R_i}} \tag{17}$$

Here, we define ΣI_i as the net ionic thermocurrent ($I_{i,net}$) and $\frac{1}{\Sigma\frac{1}{R_i}}$ as the net ionic resistance ($R_{i,net}$). In the case of a system containing monovalent cation and anion, the equation is expressed as

$$S_i = \frac{(I_+ - I_-)}{(\frac{1}{R_+} + \frac{1}{R_-})} \tag{18}$$

Finally, we can posit that $I_{i,net}$ is destructive, and $R_{i,net}$ is constructive.

Supplementary Note 2. Definition of the structural entropy

The structural entropy (ΔS_{struc}), which accounts for the changes in the structure of water beyond the hydration shell, is determined by the following equation,

$$\Delta S_{struc} = \Delta S_{hyd}^{\infty} - 0.615 S_{transl}^{\circ} - \eta S_{rot} \quad (19)$$

where ΔS_{hyd}^{∞} is the entropy gained by the hydration from vacuum to infinitely diluted water, S_{transl}° is the translational entropy loss, S_{rot} is the rotational entropy loss, and η ($0 \leq \eta \leq 1$) is an unknown numerical coefficient.[6] Both S_{transl}° and S_{rot} are negligible compared to ΔS_{hyd}^{∞}.[7]

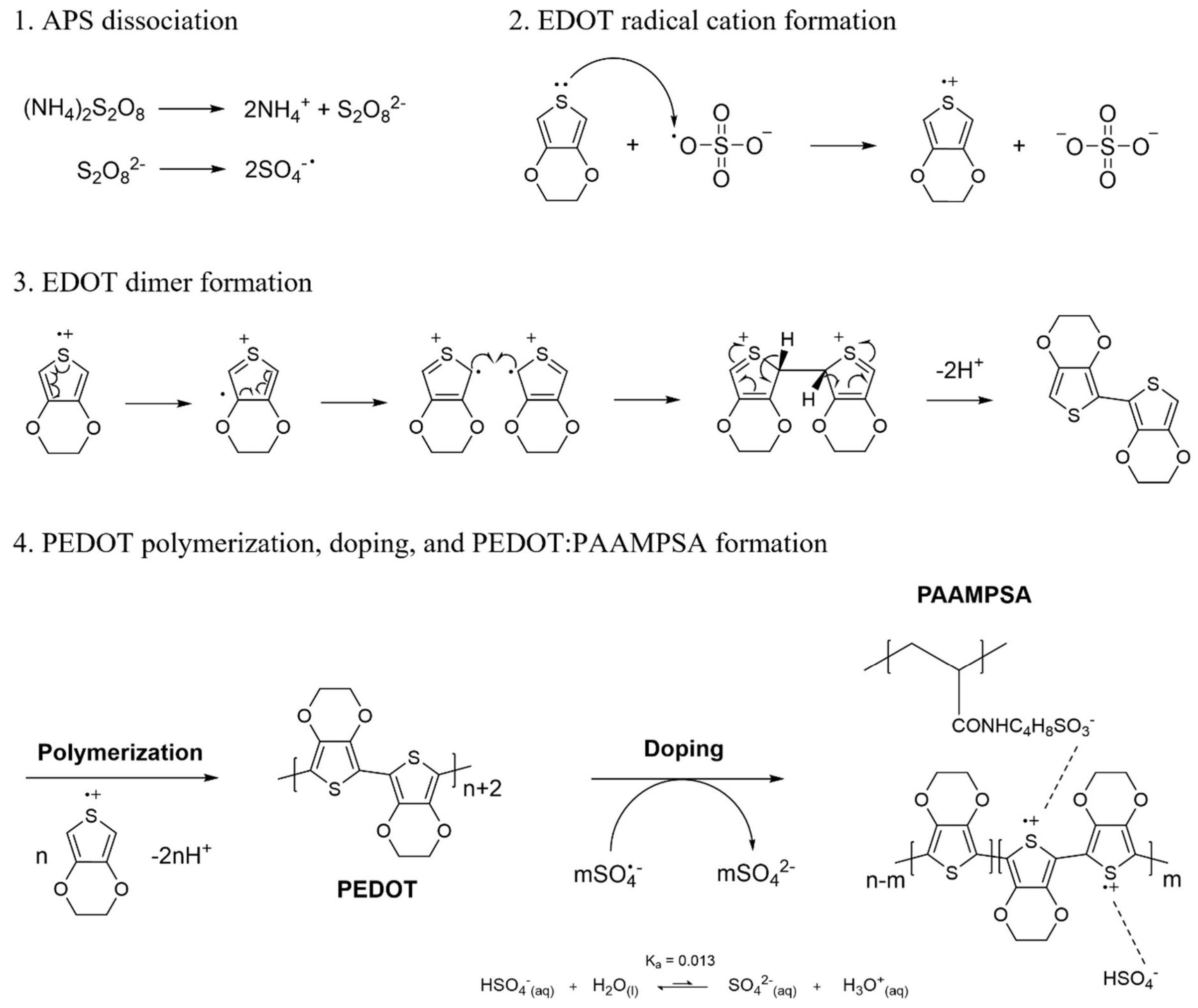

Supplementary Fig. 1 | Polymerization mechanism of PEDOT:PAAMPSA complex.

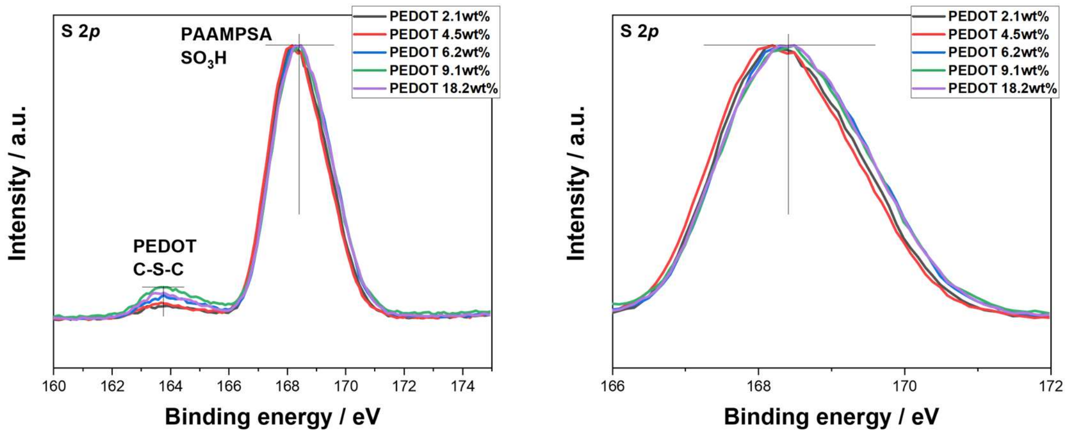

Supplementary Fig. 2 | XPS spectra (S 2*p* peak) of PEDOT:PAAMPSA films with different PEDOT/PAAMPSA ratios.

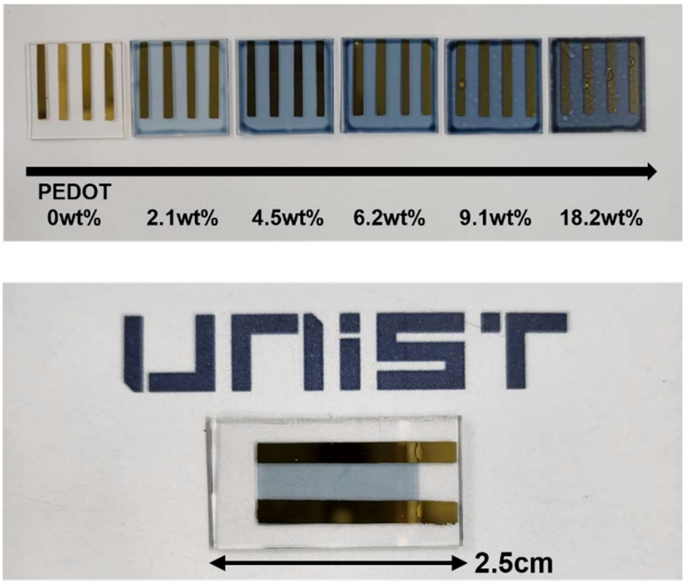

Supplementary Fig. 3 | Photos of spin-coated PEDOT:PAAMPSA:PA films with different PEDOT/PAAMPSA ratios over thermally evaporated Au electrodes on glass substrates. The films with higher PEDOT wt.% content showed darker and bluish colors due to the PEDOT aggregates.

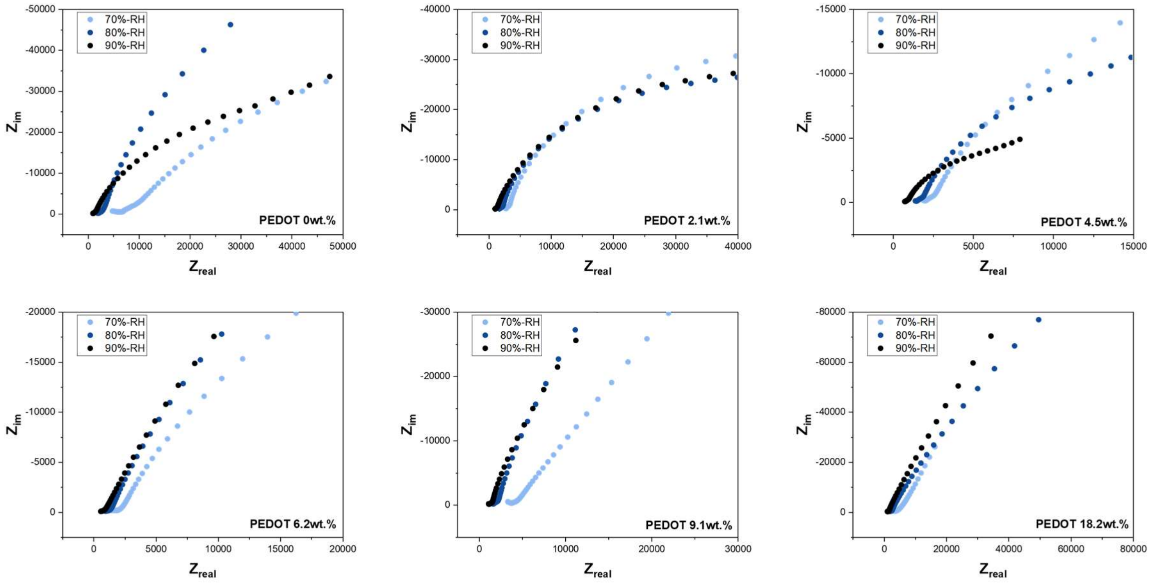

Supplementary Fig. 4 | Nyquist plots of PEDOT:PAAMPSA:PA films with different PEDOT/PAAMPSA ratios.

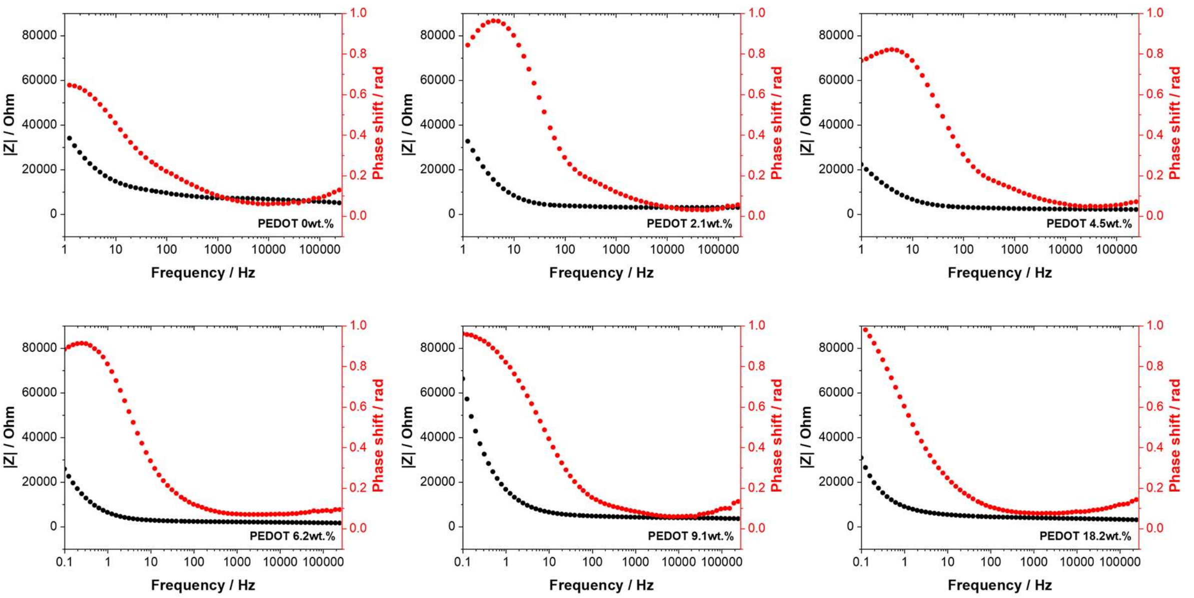

Supplementary Fig. 5 | Bode plots of PEDOT:PAAMPSA:PA films with different PEDOT/PAAMPSA ratios at 70% RH.

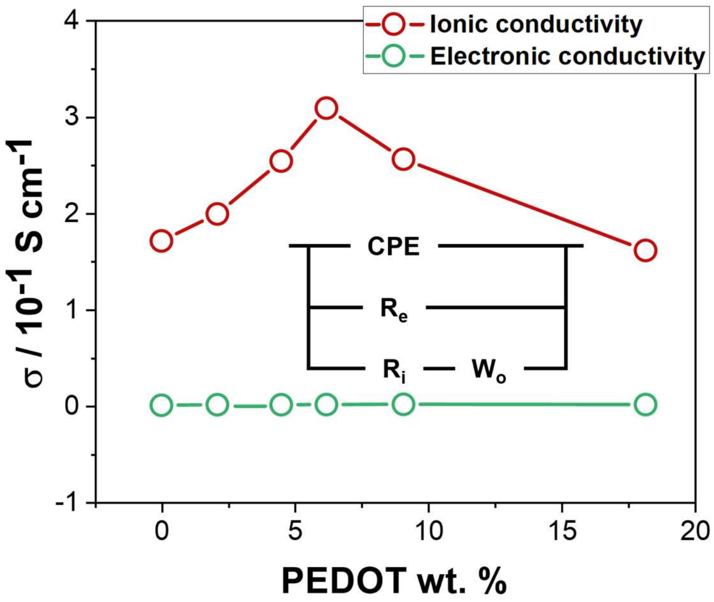

Supplementary Fig. 6 | ionic conductivity ($\boldsymbol{\sigma_i}$) and electronic conductivity of PEDOT:PAAMPSA:PA with different PEDOT/PAAMPSA ratios at 70% RH. The equivalent circuit in the figure was used to determine electronic conductivity from EIS measurement.

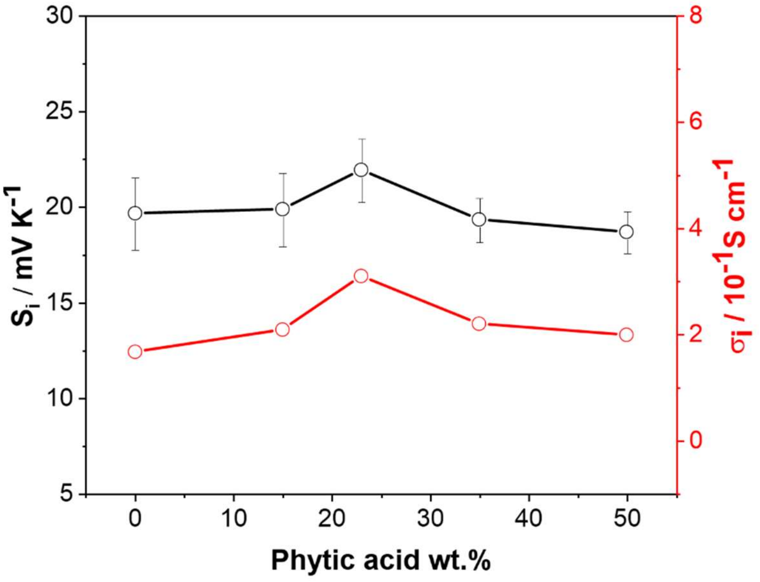

Supplementary Fig. 7 | $\boldsymbol{S_i}$ and $\boldsymbol{\sigma_i}$ of the PEDOT:PAAMPSA:PA (6.2wt.% PEDOT) film with various PA content at 70% RH.

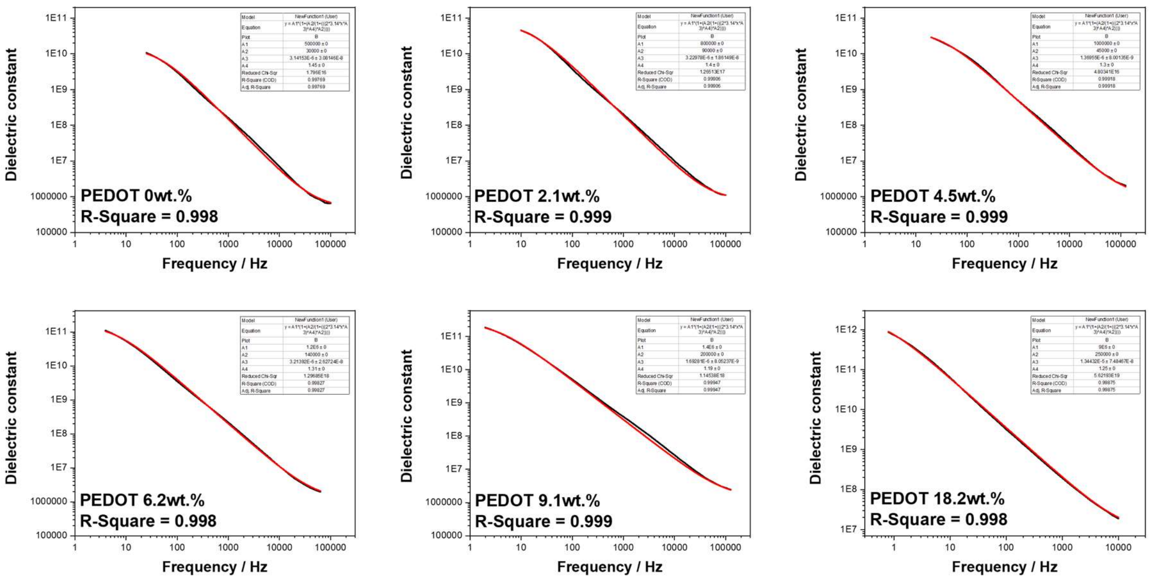

Supplementary Fig. 8 | Fitted curves (Red line) of the dielectric constant (Black line) of PEDOT:PAAMPSA films with different PEDOT/PAAMPSA ratios at 80% RH.

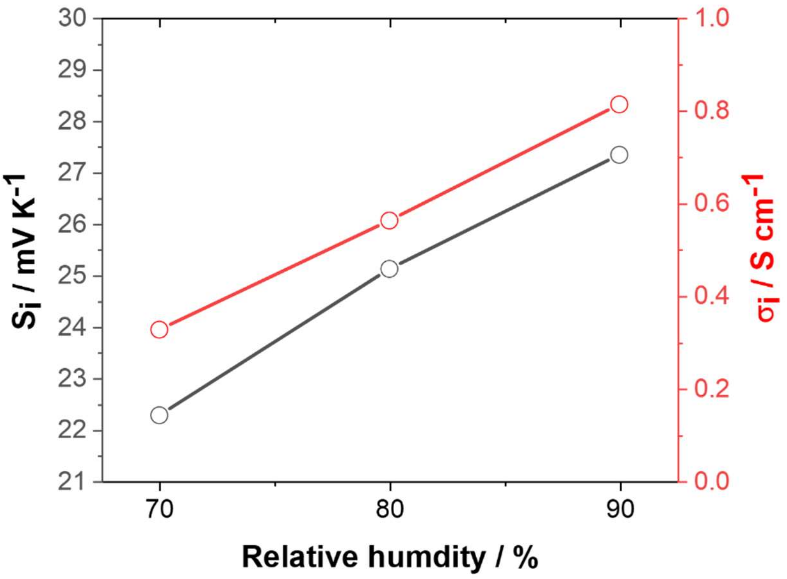

Supplementary Fig. 9 | $\boldsymbol{S_i}$ and $\boldsymbol{\sigma_i}$ of the PEDOT:PAAMPSA:PA (6.2wt% PEDOT) film at different RH values.

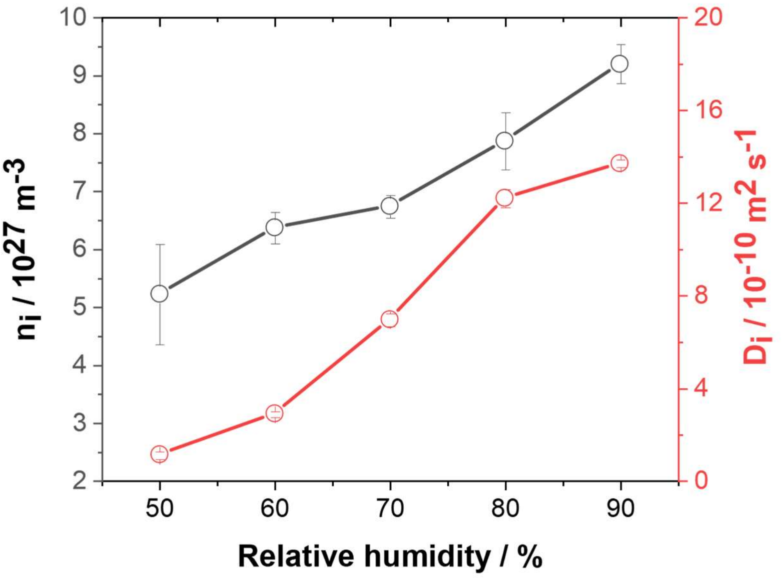

Supplementary Fig. 10 | Net $\boldsymbol{n_i}$ and $\boldsymbol{D_i}$ of PEDOT:PAAMPSA:PA (6.2wt.% PEDOT) film at different RH values.

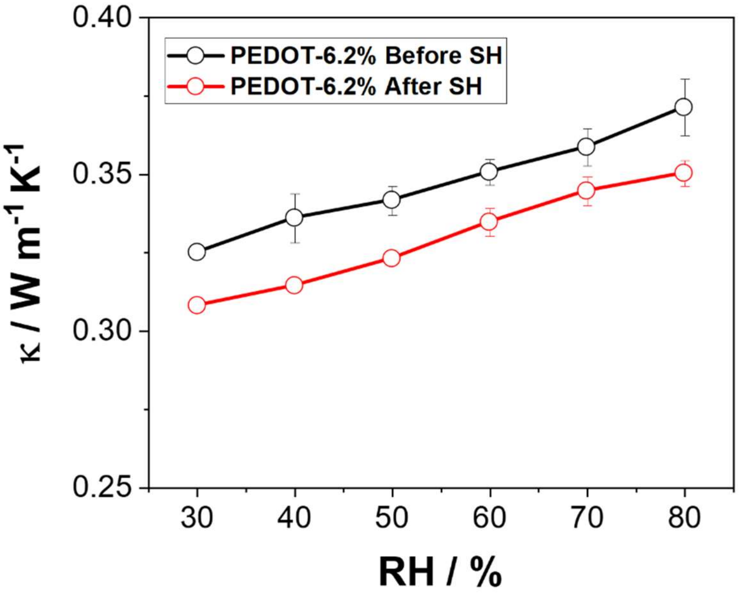

Supplementary Fig. 11 | Thermal conductivity (***κ***) of the PEDOT:PAAMPSA:PA (6.2wt.% PEDOT) film with different RH values.

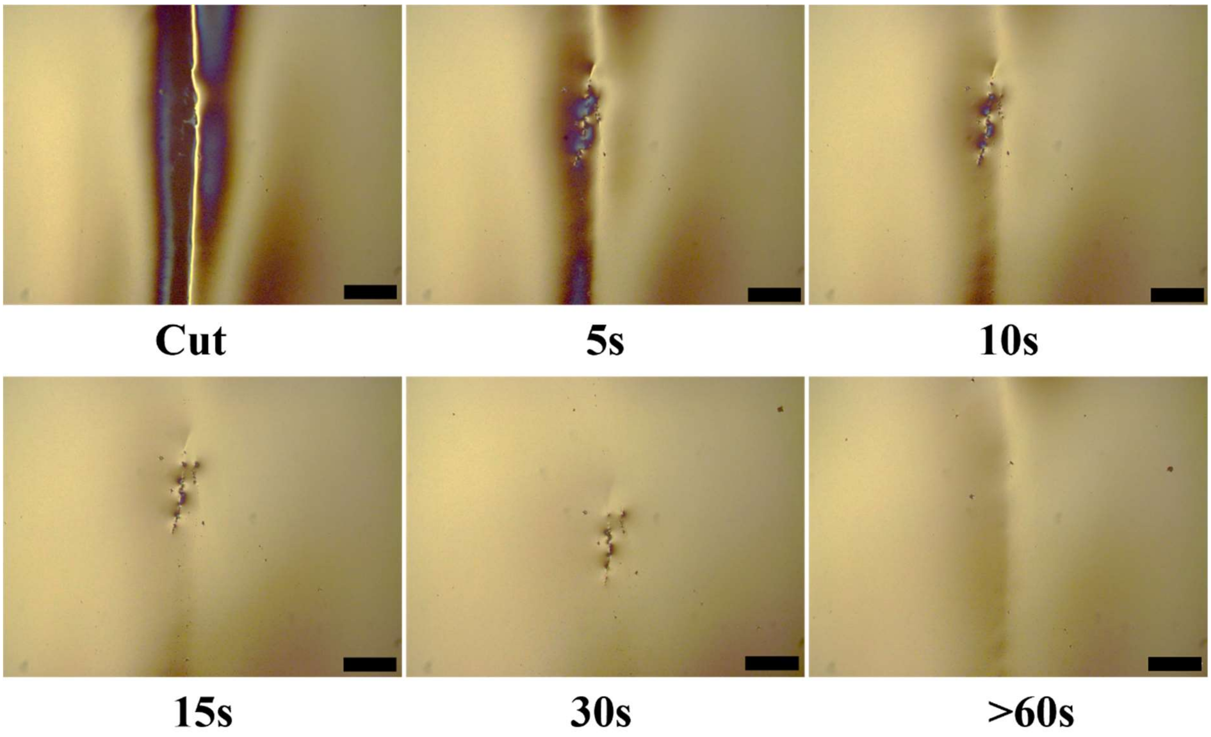

Supplementary Fig. 12 | OM images of the PEDOT:PAAMPSA:PA (6.2wt.% PEDOT) thin film during a self-healing process at 70% RH. Scale bars indicate 200 μm.

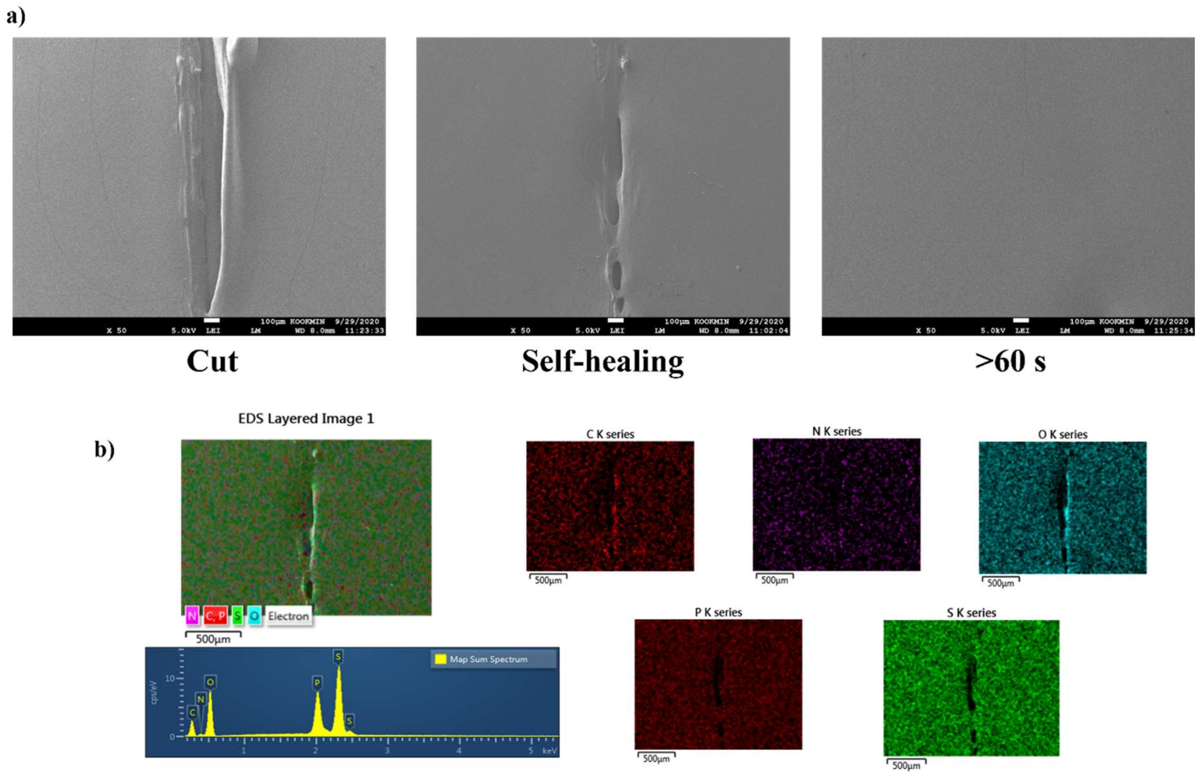

Supplementary Fig. 13 | (a) SEM and (b) EDS mapping images of the PEDOT:PAAMPSA:PA (6.2wt.% PEDOT) thin film on a glass substrate during a self-healing process at 70% RH. The SEM images in (a) are the same images in Fig. 4b.

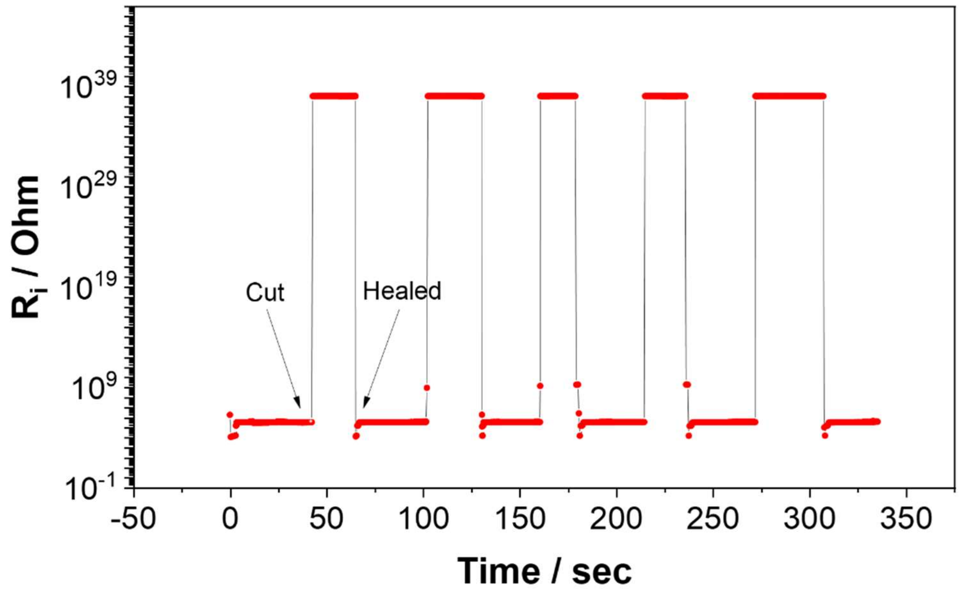

Supplementary Fig. 14 | Ionic resistance profile of the PEDOT:PAAMPSA:PA (6.2wt.% PEDOT) film during real-time self-healing cycles at 70% RH.

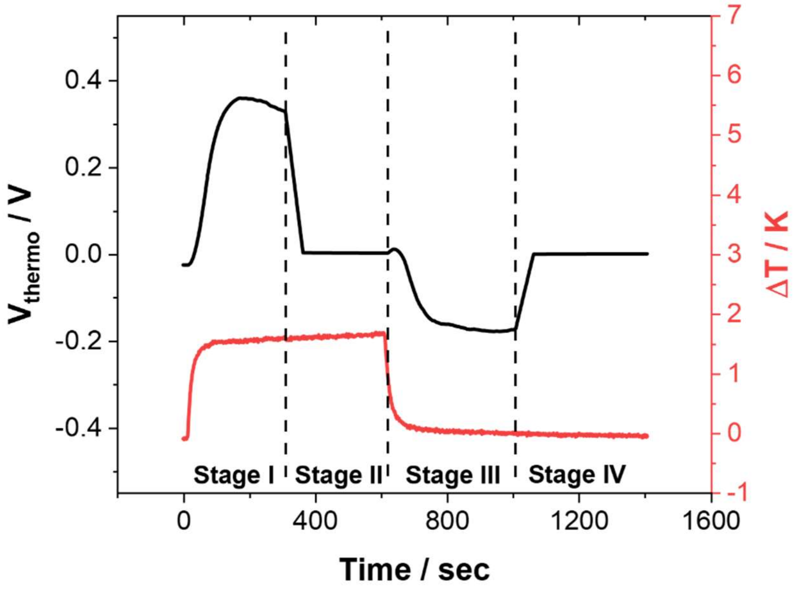

Supplementary Fig. 15 | Thermovoltage profile of the ITEC module during ITEC operation at 80% RH. A 10 kΩ load resistor was connected for charging and discharging in stage II and IV.

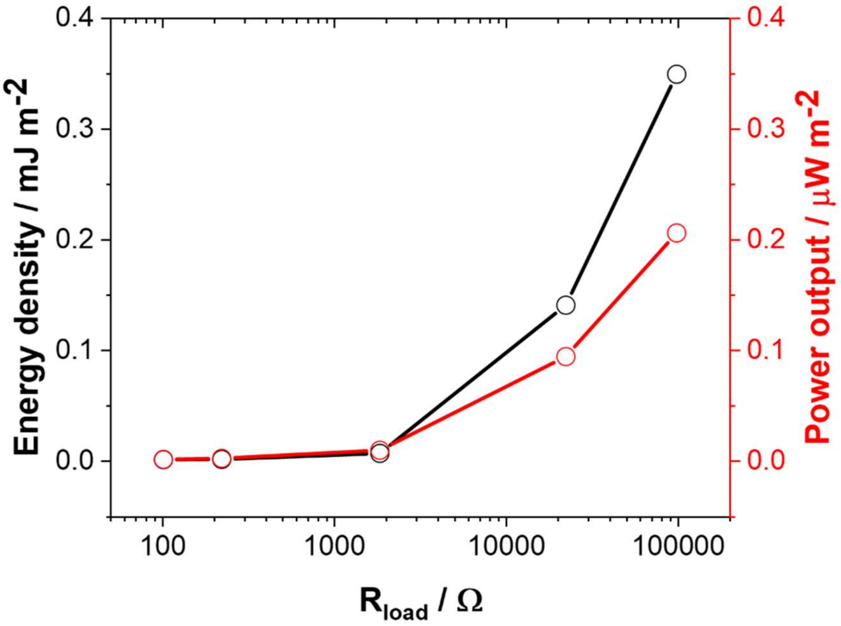

Supplementary Fig. 16 | Energy density and power output of the ITEC module with different load resistors at 80% RH.

PEDOT wt.%	pH (Method 1)	PEDOT wt.%	pH (Method 2)
0	2.19	0	2.19
2.1	2.06	2.1	2.01
4.5	2.00	4.5	1.93
6.2	1.99	6.2	1.90
9.1	1.91	9.1	1.81
18.2	1.80	18.2	1.73

Supplementary Table 1 The pH of the PEDOT:PAAMPSA aqueous solution with different PEDOT/PAAMPSA ratio. To prepare the solution, the same weight of PEDOT:PAAMPSA dispersion (666mg) was diluted with 40 mL of deionized water in Method 1 and a different weight of the PEDOT:PAAMPSA dispersion was diluted with 40 mL of deionized water to produce an equivalent molar concentration of AAMPSA (2-acrylamido-2-methyl-1-propanesulfonic acid) unit (0.012M) in Method 2.

Materials	Humidity / %	$\lvert S_i \rvert$ / mV K^{-1}	σ_i / S cm^{-1}	ZT_i	Maximum strain / %	Self-healability	Reference
**This work (PEDOT/PAAMPSA/PA)**	70	21.9	0.309	12.3	1050	Yes	
	80	25.1	0.562	28.5			
**PVA/PEDOT/PAMPS**	80	25.0	0.159	7.2	400	Yes	[8]
**PVDF-HFP/EMIM:DCA (doped)**	85	43.8	0.019	6.1	n/a	n/a	[9]
**PVDF-HFP/EMIM:OTf/FS**	90	38.3	0.011	2.34	500	Yes	[10]
**PVDF-HFP/EMIM:DCA**	70	25.4	0.018	1.8	n/a	n/a	[11]
**PEDOT/PSS/$CuCl_2$**	80	18.2	0.053	1.54	n/a	n/a	[12]
**WPU/EMIM:DCA**	90	34.5	0.008	1.3	156	n/a	[13]
**PANI/PAAMPSA/PA**	90	8.1	0.237	1.04	750	Yes	[14]
**PU/EMIM:DCA/BDB**	90	31.4	0.007	0.99	300	Yes	[15]
**PSSH**	70	7.9	0.09	0.025	n/a	n/a	[16]
**Polyamide/EMIm:Tf_2N**	n/a	1.4	0.009	n/a	n/a	Yes	[17]

Supplementary Table 2 Performance summary of state-of-the-art iTE materials.[8-17]

References

1 Zhao, D. *et al.* Polymer gels with tunable ionic Seebeck coefficient for ultra-sensitive printed thermopiles. *Nature communications* **10**, 1093 (2019).

2 Aziz, S. B., Brza, M., Saed, S. R., Hamsan, M. & Kadir, M. Ion association as a main shortcoming in polymer blend electrolytes based on CS: PS incorporated with various amounts of ammonium tetrafluoroborate. *Journal of Materials Research and Technology* **9**, 5410-5421 (2020).

3 Bandara, T., Dissanayake, M., Albinsson, I. & Mellander, B.-E. Mobile charge carrier concentration and mobility of a polymer electrolyte containing PEO and Pr4N+ I− using electrical and dielectric measurements. *Solid State Ionics* **189**, 63-68 (2011).

4 Arya, A. & Sharma, A. L. Effect of salt concentration on dielectric properties of Li-ion conducting blend polymer electrolytes. *Journal of Materials Science: Materials in Electronics* **29**, 17903-17920 (2018).

5 Han, C. G. *et al.* Giant thermopower of ionic gelatin near room temperature. *Science* **368**, 1091-1098 (2020).

6 Marcus, Y. Effect of ions on the structure of water. *Pure and Applied Chemistry* **82**, 1889-1899 (2010).

7 Saha, D. & Mukherjee, A. Impact of Ions on Individual Water Entropy. *The Journal of Physical Chemistry B* **120**, 7471-7479 (2016).

8 Cho, C., Kim, B., Park, S. & Kim, E. Bisulfate transport in hydrogels for self-healable and transparent thermoelectric harvesting films. *Energy & Environmental Science* **15**, 2049-2060 (2022).

9 Liu, Z. *et al.* Giant Thermoelectric Properties of Ionogels with Cationic Doping.

Advanced Energy Materials **12**, 2200858 (2022).

10 Akbar, Z. A. *et al.* Self-Healable and Stretchable Ionic-Liquid-Based Thermoelectric Composites with High Ionic Seebeck Coefficient. *Small* **18**, 2106937 (2022).

11 Liu, Z., Cheng, H., He, H., Li, J. & Ouyang, J. Significant Enhancement in the Thermoelectric Properties of Ionogels through Solid Network Engineering. *Advanced Functional Materials* **32**, 2109772 (2022).

12 Kim, B., Hwang, J. U. & Kim, E. Chloride transport in conductive polymer films for an n-type thermoelectric platform. *Energy & Environmental Science* **13**, 859-867 (2020).

13 Fang, Y. *et al.* Stretchable and Transparent Ionogels with High Thermoelectric Properties. *Advanced Functional Materials* **30**, 2004699 (2020).

14 Akbar, Z. A., Jeon, J.-W. & Jang, S.-Y. Intrinsically self-healable, stretchable thermoelectric materials with a large ionic Seebeck effect. *Energy & Environmental Science* **13**, 2915-2923 (2020).

15 Xu, J. *et al.* Highly Stretchable PU Ionogels with Self-Healing Capability for a Flexible Thermoelectric Generator. *ACS Applied Materials & Interfaces* **13**, 20427-20434 (2021).

16 Kim, S. L., Lin, H. T. & Yu, C. Thermally Chargeable Solid-State Supercapacitor. *Advanced Energy Materials* **6**, 1600546 (2016).

17 Jia, H., Tao, X. & Wang, Y. Flexible and Self-Healing Thermoelectric Converters Based on Thermosensitive Liquids at Low Temperature Gradient. *Advanced Electronic Materials* **2**, 1600136 (2016).
