# Supplementary material for: Self-healable polymer complex with a giant ionic thermoelectric effect
Source: Nat Commun. 2023 Jun 5;14:3246. doi: 10.1038/s41467-023-38830-w (PMC10241813; doi:10.1038/s41467-023-38830-w)
Supplement: Supplementary file 3 — Description of Additional Supplementary Files [file 41467_2023_38830_MOESM3_ESM.pdf]

List and description of supplementary movies

1. Supplementary Movie 1 | Reproducible mechanical stretchability (a strain of >500%) of PEDOT:PAAMPSA:PA free-standing film.

2. Supplementary Movie 2 | Real-time OM observation of self-healing of PEDOT:PAAMPSA:PA thin film.

3. Supplementary Movie 3 | Self-healing of PEDOT:PAAMPSA:PA free-standing film.
